# O Redescobrimento do Brasil Cardiovascular: Como Prevenimos e Tratamos a Doença Cardiovascular em Nosso País

**DOI:** 10.36660/abc.20201295

**Published:** 2021-01-27

**Authors:** Letícia Rodrigues Costa, Eduardo Vasconcelos Passos, Odilson Marcos Silvestre

**Affiliations:** 1 Universidade Federal do Acre Rio BrancoAC Brasil Universidade Federal do Acre, Rio Branco, AC - Brasil

**Keywords:** Doenças Cardiovasculares/complicações, Doenças Cardiovasculares/epidemiologia, Doenças Cardiovasculares/mortalidade, Morbimortalidade, Infarto do Miocárdio, Acidente Vascular Cerebral, Prevenção e Controle, Fatores de Risco

No Brasil, as doenças cardiovasculares (DCV) são responsáveis por 27% do total de mortes, sendo a primeira causa de óbito entre os brasileiros. Essas mortes são devidas principalmente às doenças coronárias (32%), acidente vascular cerebral (AVC) (28%) e insuficiência cardíaca (IC) (18%).^1 , 2^ Em todas as cinco regiões brasileiras essas são as principais causas de óbito, muito embora, ao se comparar com as causas não cardiovasculares, o percentual das mortes por DCV é maior nas regiões mais desenvolvidas, portanto, Sul e Sudeste.^2^

Prevenir doenças cardiovasculares é evitar mortes por infarto, AVC e IC. Além das medidas não-farmacológicas, as medidas farmacológicas são eficazes e devem ser aplicadas seguindo a estratificação do risco cardiovascular e o uso de medicamentos baseados em evidências científicas. Entre os sujeitos com alto risco cardiovascular, ou seja, aqueles com maiores chances de eventos cardiovasculares nos próximos dez anos, o uso de terapias farmacológicas salva vidas.

A terapia médica otimizada promove redução de 36% na mortalidade, 27% no desfecho morte/infarto do miocárdio/AVC e melhora a qualidade de vida entre cardiopatas. Entretanto, apesar da eficácia estabelecida e comprovada em ensaios clínicos, na vida real as taxas de adesão à terapêutica são baixas, mesmo em países desenvolvidos, com apenas 40% dos pacientes usando a terapia otimizada após 5 anos do diagnóstico.^3^

O estudo REACT traz dados novos e mensagens importantes tanto para pesquisadores quanto para o dia-a-dia dos médicos brasileiros. A proposta do estudo foi documentar a prática clínica ambulatorial no tratamento de indivíduos com alto risco cardiovascular e documentá-la tanto no período inicial quanto no seguimento de 12 meses, trazendo ainda dados de adesão à terapia otimizada, os fatores relacionados à adesão e a ocorrência de desfechos cardiovasculares nessa população.^4^

O estudo incluiu cerca de 5.000 indivíduos, sendo 70% já com DCV. Muito importante que a amostra contemplou representantes de todas as regiões do país. Porém, os dados são proporcionalmente menores nas regiões mais pobres e distantes (Norte: 6,3% e Nordeste: 14,6% da amostra total do estudo), porque nesses locais mais distantes do Brasil captar informação é mais difícil, assim como manter os sujeitos da pesquisa em seguimento também é complicado, sendo tarefa árdua informar e manter a prática da medicina baseada em evidências. Logo, a primeira mensagem que o estudo REACT nos mostra são as disparidades nacionais na ocorrência das DCV e no tratamento adequado (ou não) na ponta. Isso deve ser percebido por gestores de programas de saúde e pelas sociedades médicas para implementação de programas ajustados para as disparidades entre as regiões do Brasil.

A utilização dos fármacos baseados em evidências científicas é o preditor mais poderoso de sobrevida livre de eventos cardíacos entre portadores de doença coronária.^5^ Além disso, pelo aspecto econômico, tratamento medicamentoso otimizado em pacientes com doença coronária mostrou ser custo-efetivo em coorte brasileira.^6^ No estudo REACT, ao final de doze meses, somente 24% dos sujeitos utilizavam concomitantemente antiplaquetários, estatinas e IECA, mostrando que a imensa maioria dos pacientes não estava recebendo o tratamento que aumentaria a sobrevida e economizaria dinheiro dos cofres públicos e das famílias brasileiras. Portanto, uma outra mensagem que o estudo nos trás é justamente dizer que embora a ciência tenha avançado e nos trazido certezas sobre o tratamento das DCV, a informação ainda não chegou na ponta porque as pessoas não estão tomando os medicamentos que os fariam viver mais e melhor.

Por fim, o estudo REACT ainda encontrou dados relevantes relacionados ao controle dos fatores de risco cardiovasculares e das comorbidades. Aproximadamente 10% dos pacientes que eram portadores de diabetes mellitus e hipertensão arterial não tinham sido diagnosticados mesmo apresentando marcadores diagnósticos com valores dentro da faixa patológica. Além disso, pouco mais de 20% dos sujeitos tinham o colesterol LDL na meta terapêutica para alto risco cardiovascular. Como os pacientes estavam em centros cardiológicos especializados, esperava-se maior controle dos fatores de risco e das comorbidades; isso nos alerta para um problema ainda maior, uma vez que entre sujeitos em acompanhamento por médicos da atenção primária, pode haver maior inércia na detecção e diagnóstico dos fatores de risco e na instituição de terapêutica apropriada e alcance de metas terapêuticas para essa população. É provável que o Brasil da vida real tenha números ainda piores no diagnóstico e tratamento das DCV.

Em conclusão, as terapias modificadoras de doença reduzem a morte entre aqueles com alto risco cardiovascular. Programas de melhoria de prática clínica sob a coordenação da Sociedade Brasileira de Cardiologia, incluindo capacitação profissional com envolvimento do médico não especialista (o médico da atenção primária) devem ser implantados para garantir que informações no tema sejam disseminadas e cheguem aos cinco cantos do Brasil, aumentando o uso da terapia médica otimizada e reduzindo o número de mortes por DCV.
