# Supplementary material for: O Redescobrimento do Brasil Cardiovascular: Como Prevenimos e Tratamos a Doença Cardiovascular em Nosso País
Source: Arq Bras Cardiol. 2021 Jan 27;116(1):117–8. [Article in Portuguese] doi: 10.36660/abc.20201295 (PMC8159489; doi:10.36660/abc.20201295)
Supplement: Translation

# Translation: Rediscovering Brazil: How We Prevent and Treat Cardiovascular Disease

**Authors:** Letícia Rodrigues Costa, Eduardo Vasconcelos Passos, Odilson Marcos Silvestre

**Affiliations:** 1 Brazil Universidade Federal do Acre, Rio Branco, AC - Brazil

**Keywords:** Cardiovascular Diseases/complications, Cardiovascular Diseases/epidemiology, Cardiovascular Diseases/mortality, Morbimortality, Myocardial Infarction, Stroke, Prevention and Control, Risk Factors

In Brazil, cardiovascular diseases (CVD) represent 27% of total deaths. These are mainly due to coronary heart disease (32%), stroke (28%) and heart failure (18%).^1 , 2^Although CVD are the leading cause of death in all five Brazilian regions, the percentage of deaths from CVD is higher in the more developed regions, i.e., South and Southeast.^2^

Preventing cardiovascular disease is preventing deaths from heart attack, stroke and heart failure. In addition to non-pharmacological measures, pharmacological measures are effective and should be applied following the stratification of cardiovascular risk and use of evidence-based drugs. Among subjects with high cardiovascular risk, i.e., those with the greatest chance of cardiovascular events in the next ten years, using pharmacological therapies saves lives. Optimized medical therapy promotes a 36% reduction in mortality, 27% reduction of death/myocardial infarction/stroke and improves the quality of life of patients with heart disease. However, despite the efficacy established and proven in clinical trials, in real life the adherence to therapy is low, even in developed countries, with about 40% of patients receiving optimized therapy after 5 years of diagnosis.^3^

The REACT study brings new data and important messages both for researchers and clinical practice Brazilian doctors. The purpose of the study was to document the national outpatient clinical practice in the treatment of individuals with high cardiovascular risk and to document it both in the baseline and in the 12-month follow-up, also bringing data on adherence to optimized therapy, factors related to adherence and occurrence of cardiovascular events.^4^

The study included about 5,000 individuals, 70% of whom already had cardiovascular disease. The authors included subjects from all the five regions of the country. However, the data is proportionally smaller in the poorest and most distant regions (North: 6.3% and Northeast: 14.6% of the total sample of the study), because in these parts of Brazil capturing information is hard task, as well as follow up subjects is also difficult, making it more difficult to inform and maintain high adherence to the evidence-based medicine practice. Therefore, the first message that the REACT study shows us is the national disparities in the occurrence of CVD and in the appropriate treatment (or not) at the front where primary care doctors are practicing. This must be perceived by health program managers and medical societies to implement programs adjusted to the disparities between regions in Brazil.

Using evidence-based therapies are the most powerful predictor of longer survival free from adverse cardiac events.^5^A Brazilian study in patients with coronary disease showed that optimized drug treatment is cost-effective.^6^In the REACT study, after 12-months follow up only 24% of subjects used concomitantly antiplatelet drugs, statins and ACE inhibitors, showing that the vast majority of patients were not receiving treatment that would increase survival and save money from public coffers and from Brazilian families. Therefore, another message this study brings us is precisely to say that although science has advanced and brought us certainty about the treatment of CVD, the information has not yet reached the doctor at the front.

Finally, the REACT study also found relevant data related to the control of cardiovascular risk factors and comorbidities. Approximately 10% of patients who had diabetes mellitus and hypertension had not been diagnosed even with diagnostic markers with values within the pathological range. In addition, just over 20% of the subjects had LDL cholesterol in the therapeutic target for high cardiovascular risk. As patients were in specialized cardiological centers, greater control of risk factors and comorbidities was expected; this alert us to an even greater problem, since among subjects being monitored by primary care physicians, there may be greater inertia in the detection and diagnosis of risk factors and in the institution of appropriate therapy. It is likely that real-life Brazil has even worse numbers in the diagnosis and treatment of CVD.

In conclusion, disease-modifying therapies reduce death among those at high cardiovascular risk. Clinical practice improvement programs under the coordination of the Brazilian Society of Cardiology, including professional training with the involvement of a non-specialist physician (the primary care physician) must be implemented to ensure that information on the topic is disseminated and reaches the five corners of Brazil, increasing the use of optimized medical therapy and reducing the number of deaths from CVD.
